# Bioactive Materials for Soft Tissue Repair

**DOI:** 10.3389/fbioe.2021.613787

**Published:** 2021-02-19

**Authors:** Elisa Mazzoni, Maria Rosa Iaquinta, Carmen Lanzillotti, Chiara Mazziotta, Martina Maritati, Monica Montesi, Simone Sprio, Anna Tampieri, Mauro Tognon, Fernanda Martini

**Affiliations:** ^1^Department of Medical Sciences, University of Ferrara, Ferrara, Italy; ^2^Institute of Science and Technology for Ceramics-National Research Council (ISTEC-CNR), Faenza, Italy

**Keywords:** bioceramic, biomimetic, bioglasses, soft tissue, delivery

## Abstract

Over the past decades, age-related pathologies have increased abreast the aging population worldwide. The increased age of the population indicates that new tools, such as biomaterials/scaffolds for damaged tissues, which display high efficiency, effectively and in a limited period of time, for the regeneration of the body's tissue are needed. Indeed, scaffolds can be used as templates for three-dimensional tissue growth in order to promote the tissue healing stimulating the body's own regenerative mechanisms. In tissue engineering, several types of biomaterials are employed, such as bioceramics including calcium phosphates, bioactive glasses, and glass–ceramics. These scaffolds seem to have a high potential as biomaterials in regenerative medicine. In addition, in conjunction with other materials, such as polymers, ceramic scaffolds may be used to manufacture composite scaffolds characterized by high biocompatibility, mechanical efficiency and load-bearing capabilities that render these biomaterials suitable for regenerative medicine applications. Usually, bioceramics have been used to repair hard tissues, such as bone and dental defects. More recently, in the field of soft tissue engineering, this form of scaffold has also shown promising applications. Indeed, soft tissues are continuously exposed to damages, such as burns or mechanical traumas, tumors and degenerative pathology, and, thereby, thousands of people need remedial interventions such as biomaterials-based therapies. It is known that scaffolds can affect the ability to bind, proliferate and differentiate cells similar to those of autologous tissues. Therefore, it is important to investigate the interaction between bioceramics and somatic/stem cells derived from soft tissues in order to promote tissue healing. Biomimetic scaffolds are frequently employed as drug-delivery system using several therapeutic molecules to increase their biological performance, leading to ultimate products with innovative functionalities. This review provides an overview of essential requirements for soft tissue engineering biomaterials. Data on recent progresses of porous bioceramics and composites for tissue repair are also presented.

## Introduction

The use of biomaterials for regeneration of tissues damaged by traumatic or pathological lesions is today well-established as a promising therapeutic approach, thanks to the ability of biomaterials with appropriate composition, textured structure and bio-competent mechanical performance, to instruct and guide endogenous or previously seeded stem cells toward appropriate differentiation and new tissue formation and remodeling. Particularly, great efforts are being devoted to exploiting biomaterials for many different applications in soft tissue regeneration, owing to the possibility to obtain structures mimicking relevant compositional and structural features of the target tissue thus exhibiting excellent bioactivity (Yu et al., 2019). Recent advances in nanotechnological approaches for biomaterial design and development represent significant opportunities to employ innovative scaffolds in regenerative medicine and to generate new biomaterials for applications in tissue engineering (Ramalingam et al., 2019). Nowadays, biomaterials of very different nature are investigated and tested for regeneration of soft tissues, including ceramics, bio-glasses, polymers, hydrogels, and related composites. Among ceramics, calcium phosphates and more recently, calcium silicates emerged as elective materials; particularly hydroxyapatite as nanoparticles or nanostructured bodies, exhibit excellent biocompatibility, bioresorbability, and ability to safely direct stem cell activity, so that, besides applications in the regrow of bones and teeth, nano-apatites are currently investigated for regeneration of the skin, muscles and gums (Zhou and Lee, 2011; Bordea et al., 2020).

**Figure 1:**
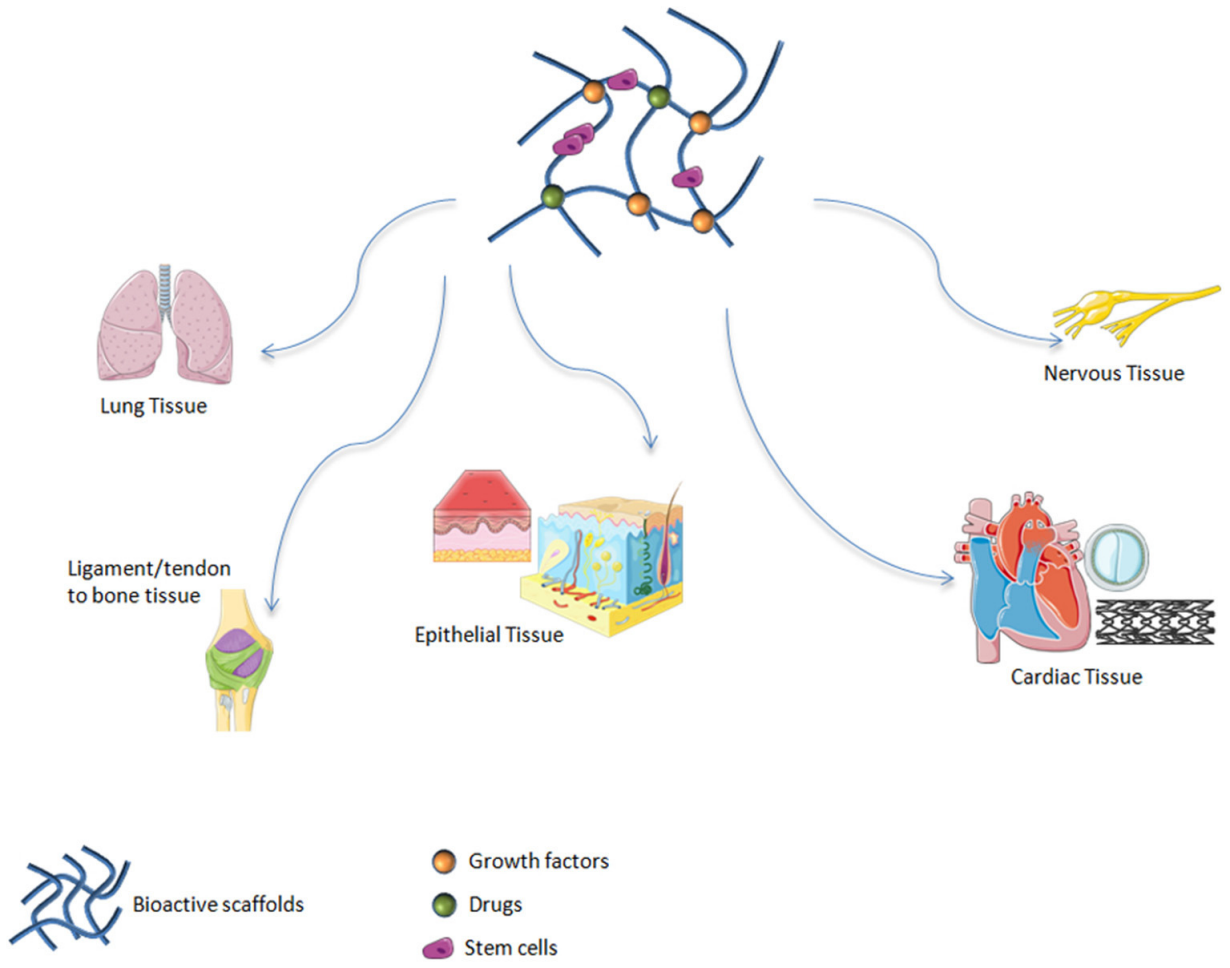
Bioactive scaffolds applications in regenerative medicine. It has been demonstrated that these materials are suitable candidates for treating soft tissue damage including lung, ligament, tendon, epithelial, cardiac, and nervous tissues.

Among inorganic materials, bioactive glasses (BGs) are widely explored in soft tissue regeneration. It has been demonstrated that these materials are suitable candidates for treating soft tissue damage including cardiac (Qi et al., 2020), lung (Kargozar et al., 2019), nervous and epithelial tissues (Kargozar et al., 2019) (Figure 1).

Polymers offer a huge variety of compounds that so far were tested for applications in medicine, particularly for soft tissue applications, as they can be easily tailor made to approach the chemical/physical/structural characteristics of soft tissues. For example, polycaprolactone (PCL) is an aliphatic polyester film-forming candidate, that is widely used in pharmaceutical and medical tools due to its bio-degradability/-compatibility and mechanical properties (Kai et al., 2017). The development of composites is made relatively easier by the nature of polymeric compounds, enabling the association of natural and synthetic polymers to develop scaffolds for both soft (Siddiqui et al., 2018) and hard tissue repair (Iaquinta et al., 2019a). In particular, composite biomaterials have been extensively studied for several application including skin tissue engineering (Chaudhari et al., 2016), nerve (Shafei et al., 2017), and cardiovascular tissue repair (Ahmed, 2013).

Hydrogels stand out among biomaterials for regenerative medicine use, thanks to their properties, which are compatible with those of biological tissues (Talebian et al., 2019; Mondal et al., 2020). Specifically, innovative materials which may constitute 3D tissue model of study are represented by different hydrogels. Indeed, their characteristics are similar to the ECM (Zhao et al., 2020). Three-dimensional self-assembled peptide hydrogels have demonstrated promising prospects in bio-related applications due to their porous structure, good mechanical stability, high biocompatibility and fast functionalization (Mondal et al., 2020). Hydrogels have come to the fore among all biomaterials as innovative scaffolds for drug delivery and cell growth in soft, such as cartilage (Zhu et al., 2018). In comparison to the other biomaterials, hydrogels can be produced with characteristics similar to soft-tissues (Wang et al., 2018).

Finally, all the above-mentioned biomaterials can be set up for *in situ* delivery of various pharmaceutical compounds. This approach, widely investigated since at least a decade, opens to new personalized therapies in regenerative medicine where the association of bioactive substrates with local delivery of therapeutics can yield enhanced effectiveness, safety in respect to the systemic drug administration.

With this, the present review offers a broad perspective of the use of different classes of biomaterials for soft tissue regeneration.

## Applications of Bioceramics in Soft Tissue Repair

Bioceramics are frequently used in medicine as scaffolds to replace or regenerate damaged tissues. In this respect, the physico-chemical properties of bioceramics such as calcium phosphates (CaPs) are effective for cell instruction and driving natural metabolism, thanks to the biomimetic composition. Early literature on the biological ability of CaPs dates back to 1920 (Iviglia et al., 2019). Since then, a considerable amount of information and data has been developed and consolidated about their chemistry, formulations and properties (Iviglia et al., 2019). Due to its composition, which resembles the inorganic bone tissue, hydroxyapatite (HA) is a material with multiple potential uses that can be developed as nanoparticles or assembled into nanostructures thus functioning as scaffolds with physical, chemical, mechanical and biological features tailored for different tissue targets, particularly for hard tissue regeneration. In spite of this prevalence, HA has been recently explored also for purpose of enhanced skin care, particularly when employed as nanoparticles embedded in bio-organic matrixes, where the high dilution of the inorganic phase can help to achieve bio-stimulatory effects improving skin quality, firmness and rejuvenation, as well as sustaining skin regeneration upon damages derived from exposure to sun, from contact with various harmful chemical substances or due to acne (Antonino and Francesco, 2021). In this respect it was found that HA can act as excellent skin barrier, particularly useful when the protective *stratum corneum* results damaged and provide insufficient ability to contrast harmful environmental effects or water loss (Gaudinat, 2007). Previous studies highlight that a basis for the effectiveness of HA in skin healing is given by the affinity between HA and the natural skin collagen as well as the progressive bio-dissolution of HA nanoparticles into its Ca^2+^ and ${\text{PO}}_{4}^{3-}$ ions constituents provide a safe sustain to skin collagen neo-formation and recovery of functional and aesthetic features without any adverse effects, so that HA is today FDA-approved for many applications in skin care (Antonio and Arroyo Trídico, 2019; de Almeida et al., 2019).

Besides skin regeneration, CaPs are tested also for regeneration of periodontal tissues. Developmental factors and signaling molecules depend on periodontal regeneration, and less on the pulp-dentinal complex, in some research. eta-tricalcium phosphate bioceramic (β-TCP) has been combined with human stem cells derived from periodontal ligament (PDLSCs-CA) and umbilical cord (hUCMSCs-CA), respectively. Their regenerative potentials have been investigated *in vivo* in a rat model of study for inflammatory periodontal disease (Shang et al., 2017). Interestingly, hUCMSCs as well as hPDLSCs regenerated in inflammatory conditions soft and hard periodontal tissues (Shang et al., 2017).

A different class of bioceramics emerging for tissue regeneration purposes are calcium silicates (CS); this class of bioceramics include a wide range of compositions, including also natural minerals, which can be obtained by lab synthesis, by reaction of calcium salts with silica (Li et al., 2020). The presence of calcium-rich surface sites and of silica confer to calcium silicates the ability to establish tight bonds with surrounding tissues, particularly when bone regeneration is addressed (Wan et al., 2005). On the other hand, the ability of ${\text{SiO}}_{4}^{4-}$ ions release during CS degradation provide biologic effects that can be suitable also for the healing of soft tissues, particularly for skin regeneration, such as stimulation of the angiogenesis (Yu et al., 2019) and support to the synthesis and deposition of collagen type I (COL-I) (Reffitt et al., 2003). In extensive cutaneous vascular damage cases, the skin tissue loses the self-renewal capacity, as shown in diabetic wounds where the angiogenesis activity is reduced (Conway et al., 2001). In this scenario bioceramics silicate favors the tissue repair. The study conducted by Li et al., proved that CS promotes angiogenesis favoring vascular endothelial growth factor (VEGF) and VEGF receptor (VEGFr) expressions in human dermal fibroblast (HDF) and human umbilical vein endothelial cell (HUVEC) (Li and Chang, 2013). Li et al. investigated the potential activity of CS in the cure of diabetic wound on High-Glucose (HG)-inducted fibroblasts. They confirmed that CS improves wound healing process stimulating proliferation, migration and differentiation of cells (Li et al., 2020). Another important aspect of CS is the presence of Ca^2+^ ions, that released in the environment cause an increase of pH leading to acquisition of antibacterial activity (Yu et al., 2019). CS seems to play an important role, not only for skin repair but also for the cure of myocardial necrosis, articular cartilage, adipose tissue and neovascularization growth. Further investigations are needed to know all applications of CSs and their mechanisms of action after the implantation in the injured tissue.

## Bioactive Glasses to Repairsoft Tissue Injuries

**Figure 2:**
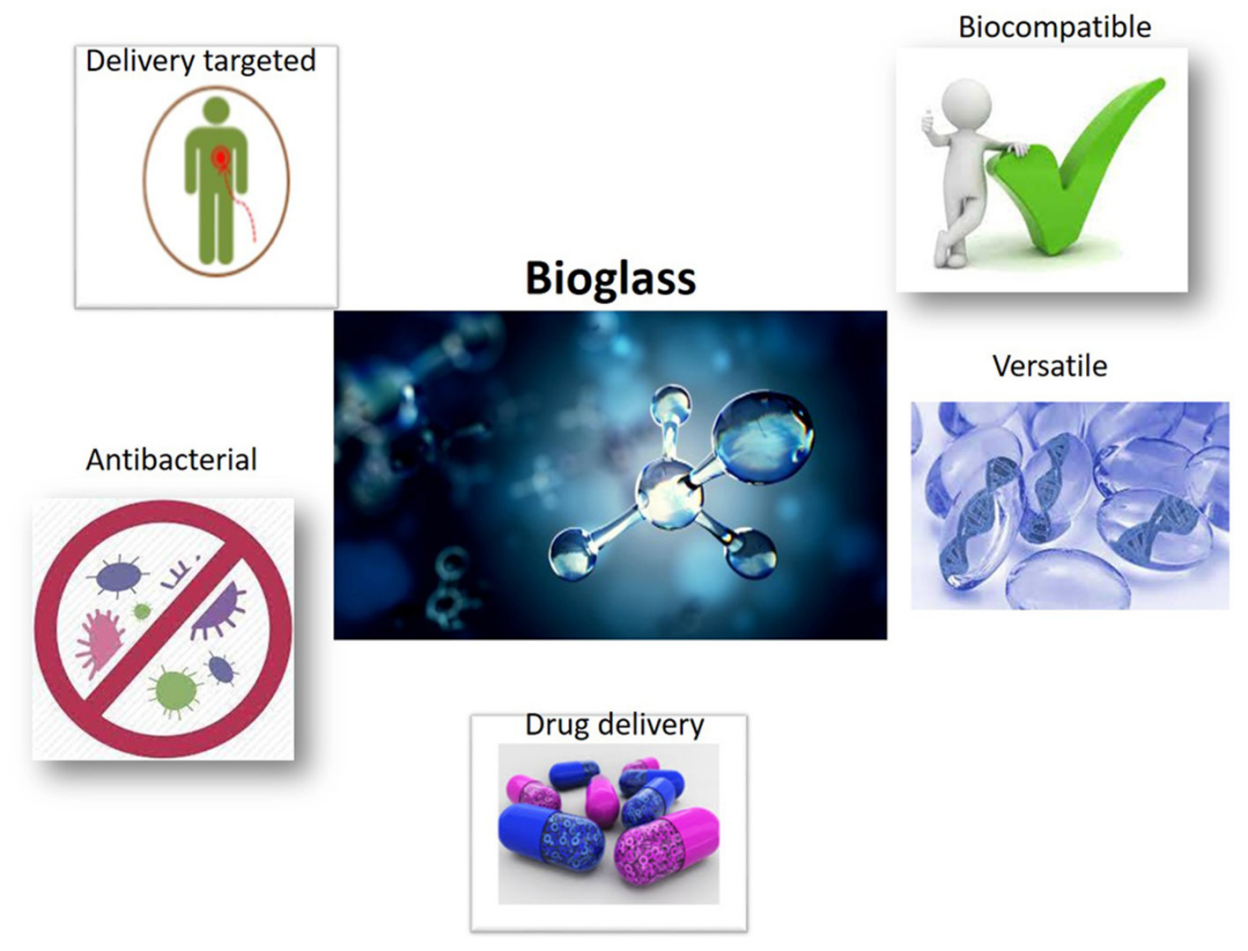
Properties of bioactive glasses BGs as scaffolds employing in regenerative medicine. The principal proprieties of BGs are an optimal drug delivery, antibacterial and biocompatible proprieties, and versatile use.

BGs are a class of biomaterials firstly developed by Hench et al. (1971). These authors designated the 45SiO_2_-24.5Na_2_O−24.5CaO−6P_2_O_5_ (wt%) composition, among various glass preparations based on the SiO_2_-Na_2_O–CaO–P_2_O_5_ oxide system. This BG is known as 45S5 Bioglass®. Molecules in the selected composition make the biomaterial surface very reactive in the biological environment. 45S5 Bioglass® is the first model of third generation biomaterial, namely biomaterial, which presents biocompatibility, an improvement in growth of contact tissue and genetic activation of specific cell pathways (Baino et al., 2018). BGs are generally created by melting-quenching methods, or by sol-gel techniques. Glasses derived from melting-quenching can be dispensed into shapes to create components of various sizes and shapes, i.e., rods and bars. BGs can also be fray to produce glass fibers, which are also promising in soft tissue engineering, while glass powder can be used as a starting material to fabricate three-dimensional (3D) porous scaffolds. It is possible to produce glasses, through wet synthesis, with mesoporous textures that can be used for local drug delivery (Baino et al., 2016; Kargozar et al., 2019) (Figure 2). BGs can be divided into different groupings, depending on the representative oxide system, such as the silicate system (SiO_2_-based), borate system (B_2_O_3_-based) and phosphate system (P_2_O_5_-based) (Baino et al., 2016). BGs, or rather, phosphate system BGs, are resorbable, whereas the dissolution process of BGs in the biological environment takes place upon contact with body fluids (Kargozar et al., 2019). Metal ions released from the BGs' structure have distinct therapeutic functions, such as cell proliferation, stimulation of angiogenesis, as well as anti-inflammatory and antibacterial activities (Kargozar et al., 2018). The main ions, which are responsible for cell proliferation and tissue reconstruction acceleration, are Sr^2+^, Si^4+^, and Ca^2+^. Ions-like Cu^+^, Cu^2+^, Co^2+^, B^3+^, Zn^2+^ are able to improve angiogenesis; Ag^+^ and Zn^2+^ have anti-inflammatory properties; Cu^+^, Cu^2+^, Ag^+^, Ga^3+^, Zn^2^ produce an antibacterial effect via the increase of local pH and ROS production (Kargozar et al., 2018).

Depending on their structure, BGs can be mesoporous, containing pores with diameters between 2 and 50 nm. Mesoporous BGs (MBGs) have been considered third generation BGs, while the first MBGs were synthesized by (Yan et al., 2004). MBGs are appropriate vehicles for the release of different drugs and growth factors, which can be located in the mesoporous. Other types of BGs are MBGs microspheres (MBGMs) produced by Ostomel et al. (2006), which are able to increase surface area, resulting in accelerated HA deposition when immersed in a body fluid. This characteristic has been important for its success in tissue engineering applications (Ostomel et al., 2006). Different commercial formulas of BGs have been clinically employed since 1993, to fill periodontal bone defects, for orthopedic and dental applications, in middle ear surgery and in one of the first prototypes of cochlear implants (Baino et al., 2016). Bioglasses have interesting advantages compared to HA, including their binding capability to hard and soft tissue. Indeed, HA is limited in its binding activity, i.e., it binds only to hard tissues. Moreover, HA needs an additional tool to keep implants *in situ* (Padrines et al., 2000). In the last 50 years, since the systemization of BGs by Hench, typical applications of BGs include those listed above, whereas more recently many studies demonstrated the suitability of BGs in soft tissue engineering.

### Bioglasses for Cardiac Tissue Regeneration

Myocardial infarction (MI), consisting in the blockage of coronary arteries and subsequent tissue death, is the cause of many deaths worldwide (Gerber et al., 2016). Difficulties in medical intervention and the inability of cardiomyocytes to regenerate have led to the need to find alternative methods of repair for heart tissue injuries. Therefore, tissue engineering using injectable scaffolds and cardiac reinforcements have been analyzed as efficacious instruments to repair damaged cardiac muscle (Rane and Christman, 2011). In this scenario, BGs have been considered as appropriate for cardiac tissue renewal in the form of nanoparticles placed on soft matrices. Polymeric heart patches were developed by Chen et al. (2008), as heart mechanical support and as cell delivery systems to repair damaged tissue. In order to accomplish these two intents, Chen's research team produced different elastomeric nanocomposites composed of soft elastomer (poly[glycerol sebacate] PGS) and nanoparticle of 45S5 Bioglass® with concentrations ranging from 0 to 10 wt %. PGS gives polymer heart patches mechanical flexible support, while nano-sized BGs give both mechanical properties and an anchor point for cells releasing into the surrounding physiological environment. Furthermore, glass incorporation in the polymer was found to reduce PGS-nanoBioglass® scaffoldacidity and improve biocompatibility (Kargozar et al., 2017). *In vitro* studies conducted on human endometrial stromal cell (ESC)-derived cardiomyocytes (hESC-CM) confirm enhanced biocompatibility for PGS-nano-Bioglass® compared to PGS without glass nanoparticles. Moreover, upon observing cells in contact with PGS-nano-Bioglass® and cells in the control group cultured in standard medium, cellular functional activity was found to be similar in the two experimental groups. BGs are linked to ions that stimulate cells to secrete angiogenic factors and cytokines allowing invasion of the engineering scaffolds from the vessels. In this context, it is important to recall studies by Barabadi (Barabadi et al., 2016) that synthesized gelatin-collagen hydrogel (Gel/Col) containing BGs (Gel/Col/BG) (Figure 1) in order to solve the lack of functional vessels in tissue engineering field. Results demonstrated an increased expression of the vascular endothelial growth factor (VEGF) (Barabadi et al., 2016). We can conclude that nano-Bioglasses have been proven to be suitable candidates in treating heart failure and for regenerating cardiac tissue through the cardiac patch strategy.

### Bioglasses for Pulmonary Tissue Regeneration

Lung tissue presents poor regenerative capabilities. This characteristic represents a major problem for patients suffering from severe chronic obstructive pulmonary disease (COPD), pulmonary hyperextension, and cystic fibrosis who are consequently forced to undergo lung transplants (Petersen et al., 2010). In this scenario, tissue engineering using BGs has been considered. A pioneer study was conducted by Tan et al., on murine lung epithelial cells (MLE-12) to test the biocompatibility of sol-gel 58S glass scaffolds containing amine or mercaptan groups or laminin. Results demonstrated that lung cells colonized all types of scaffold, which is therefore considered biocompatible and very effective for improving cell adhesion and growth (Tan et al., 2003). In a further study conducted on human lung-derived immortal cells, Verrier et al. tested foam based on porous poly (DL-lactic acid) (PDLLA) scaffolds combined with 45S5 Bioglass®. *In vitro* assays showed an enhancement in cell adhesion and proliferation, depending on foam BG content. The greatest cell proliferation was detected on cells seeded on scaffolds containing glass at 5 wt% (Verrier et al., 2004). Despite the positive results, further studies are necessary to understand lung cell proliferation/differentiation and to select appropriate glass compositions, morphologies and structures before considering BGs in lung tissue engineering.

### Bioglasses for Nervous Tissue Regeneration

Peripheral nerve injuries cannot always be treated by surgery, whereas in some cases injuries could lead to partial or total loss of nerve functions and sensory deprivation in the surrounding area. Nowadays, autografts represent the gold standard treatment for healing nerve damage even if these methods may present collateral effects. Alternative routes should be considered for repairing nervous tissue damage. To this end, BGs represent a valid alternative for nervous tissue engineering. The first *in vivo* study regarding BGs was conducted to repair the sheep facial nerve. Investigators developed a resorbable phosphate glass tube, whereas they were implanted in the epineurium through holes at the ends of the tube. Three months later it was observed that the glass tube was completely dissolved and that the nerves were entirely regenerated (Gilchrist et al., 1998). Until now, the nerve guidance channel (NGC) has been used in surgery as a transplantation technique. This device is a canal sutured between the two nerve stumps protecting the regenerating nerve from tissue infiltration and providing the maximal accumulation of soluble factors. Recently, the NGCs have been improved with the addition of BGs in the form of nano-metric-fibers or micrometric diameters in order to guide Schwann cell migration and axonal growth during nerves regeneration. The use of Bioglass® caused a re-innervation analogous to auto-transplant treatment (Daly et al., 2012). In order to give direction to the growth and arrangement of nerve cells, Bunting et al. (2005) developed a scaffold using silastic ducts in rats, by introducing 45S5 Bioglass® into the fibers. This study was conducted with 4 experimental groups (i) rats having ducts with BG's fibers; (ii) rats with empty ducts; (iii) rats with nerve autograph; and (iv) rats that underwent nerve excision. Nerve tissue regeneration was similar in groups (i) and (iii). Remarkably, nerve tissue regeneration was higher in the rats from group (i), then (ii), and (iv) (Bunting et al., 2005). Furthermore, Kim et al., conducted *in vitro* and *in vivo* studies using different approaches for neuronal guidance constructs. These studies involved laminated phosphate glass microfibers (PGfs) in the compact collagen, then testing their performance by spreading dorsal root ganglion (DRG) cells from mature rats on the created ducts. Subsequently, these were tested *in vivo* by implanting the DRG cells on adult rats. Nerve conduits with PGfs showed an increase in neurite growth and faster rehabilitation of motor control *in vivo*. Histological results on the tested conduits engraft in the exported sciatic nerve demonstrated axonal expansion forward the support compared to the control group (Kim et al., 2015). A further kind of conduit, nano-bioactive glass/gelatin (BGGC) phosphate-based ducts were produced by research group of Koudehi et al. (2014) to regenerate peripheral nerve in rats with severed sciatic nerves. Rates of nerves regeneration and muscle contractility were statistically equivalent in BGGC treated rats compared to the control group (Koudehi et al., 2014).

Important damage of the nervous system is represented by spinal cord injury, which could additionally pose severe problems, such as neurological deficits and a lack of sensory and motor functions. Nowadays, treatment consists in surgery stabilization of the spinal cord, drug therapy and rehabilitation, which can stimulate the plasticity of the spinal cord. Several studies, reported below, have been performed in this field in order to discover devices for the treatment of spinal cord injury. *In vivo* study, by Joo et al. (2012), attempted to demonstrate the use of collagen/phosphate glass fiber constructs for treating this kind of nerve damage. The study obtained promising results after implanting collagen/phosphate glass fibers in divided spinal cord. Indeed, 2 months after surgery, fibers showed an improvement in terms of locomotion and bladder functions compared to pure collagen scaffolds, whereas axonal growth along the stumps was identified only in the containing scaffold fibers (Joo et al., 2012).

Previous results obtained by some investigators have suggested that the use of BGs in nerve tissue engineering is promising since it presents an improvement in nerve damage regeneration and repair, which is significantly higher compared to traditional scaffolds or care, while at the same time they allow axonal growth in the employed cells. However, some adjustments in emerging BGs are needed with regards to the amount of the chemical composition and the type of material to be employed for tubular conduits. It is important to investigate the outcome of different types of glasses like silicate, phosphate and borate glasses on target cells/tissues.

### Bioglasses for Epithelial Tissue Regeneration

Epithelial tissues are the pivotal structures covering the whole body skin, the external surfaces and internal cavities of organs, such as the upper respiratory, gastrointestinal, and reproductive tracts (Rotondo et al., 2015; Torreggiani et al., 2019; Malagutti et al., 2020). Damage/disease of the epithelial tissue is a serious problem (Mazzoni et al., 2016; Rotondo et al., 2016, 2017, 2018; Corazza et al., 2020; Preti et al., 2020). Indeed, epithelial tissue is responsible for protection, absorption, secretion, and transport in the body. To date, given the importance of epithelial tissue, new biomaterials responsible for repairing epithelial tissue injury have been tested. Among these biomaterials BGs are the most promising scaffolds because of the antibacterial activity of their ions which prevent infection in the wound sites. Nano-fibrous BG dressing and ointments containing BGs particles were successfully employed in clinical studies on wound healing (Miguez-Pacheco et al., 2015). Based on chemical composition, BGs have been considered scaffolds suitable to accelerate wound healing process. Indeed, BGs seem to stimulate gene expression of genes involved in the healing process, such as vascular endothelial growth factor (VEGF), basic fibroblast growth factors (bFGF) and vascular cell adhesion protein (VCAM) and the capability of 45S5 Bioglass® to protect endothelial cells and enhance gap junctions accelerating wound healing (Kargozar et al., 2019). Li et al., demonstrated the potential of 45S5 Bioglass® ion extracts to promote wound healing process in an *in vitro* and *in vivo* studies by stimulating the formation of gap junction connexin 43 (Cx43), a protein that takes part in gap junction intracellular communication. The *in vitro* experiment showed that after 7 days of incubation of endothelial cells with 45S5 Bioglass® ion cell death was decreased, whereas an overexpression of VEGF, bFGF, and Cx43 protein was registered. Subsequently, *in vivo* experiments conducted on rats demonstrated an increased expression of Cx43 and vascularization, together with an augmented wound healing (Li et al., 2016). The most highly-studied BGs in the context of wound healing are silicate-based glasses that lead to an increase in the local pH and antibacterial effect. These characteristics are due to the release of Si^4+^, which is also responsible for stimulating collagen production and neovascularization (Xu et al., 2015).

More recently, Xie et al., have demonstrated that the sol-gel derived silicate-based BG known as 90S [(90)SiO_2_-(6)CaO-(4)P_2_O_5_ (mol %)], when in direct contact with fibroblast, downregulates transforming growth factor-beta (TGF-β) signaling pathway, with consequences in the proliferation and tissue remodeling stage (Xie et al., 2016). Additionally, 90S BG seems to regulate of collagen types I and III, fibronectin and alpha-smooth muscle actin (α-SMA) expression, by preventing fibroblast differentiation. Another study concerning silicate-based BGs grafted in bioactive skin has shown that ionic dissolution stimulates fibroblasts to secrete growth factors for healing processes leading to wound closure (Yu et al., 2016). Moreover, an *in vivo* study showed that the presence of calcium nanoparticles on silica enhance wound closure since Ca^2+^ leads to an acceleration in blood coagulation, an increase in fibroblast proliferation and collagen concentration (Kawai et al., 2011).

As well as silicate, borate-based glasses exhibit great expectations in wound healing implementation due to their high dissolution degree, rise in local pH and the ability of boron to stimulate angiogenesis (Naseri et al., 2017). In a previous study, a resorbable borate melt-derived BG named 13-93B3 was produced with following composition: P_2_O_5_ (3,7) B_2_O_3_ (56,6) Na_2_O (5,5) K_2_O (11,2) CaO (18,5) MgO (4,6) (wt%) (Kargozar et al., 2019). BG 13-93B3 fibers mimic the structure of the fibrin clot inducing fast wound closure in animals and diabetic patients, who did not respond to traditional therapy. 13-93B3, because releases Ca^2+^ ions, stimulates angiogenesis, induces epidermal cell migration and accelerate skin regeneration. In addition, 13-93B3 glass which received approval by the Food and Drug Administration (FDA) is successfully commercialized for veterinarian medicine as “RediHeal” for skin repair in animals (Naseri et al., 2017). Permission by the FDA for clinical use in humans is pending (Kargozar et al., 2019).

A further consistent *in vivo* study was carried out by Lin et al. These researchers produced Vasiline-based compounds with sol-gel bioactive glass 58S (SGBG-58S), nanoscale bioactive glass (NBG-58S) and melt-derived 45S5 powder and tested them on the wounds of healthy and diabetic rats for 16 days. At the end of the experiment, results highlighted that in both healthy and diabetic rats the healing was enhanced by BGs, specifically SGBG-58S which presented almost complete wound healing; as expected, in diabetic rats the wound healing process was longer than in healthy rats (Lin et al., 2012).

In addition to wound healing, BGs are also a point of interest for the repair of other epithelia, such as the respiratory and gastrointestinal epithelium. The respiratory epithelium is composed of ciliated cells that confer moisture and protection to the airways. Respiratory tissue damage is currently difficult to cure, being a different lung transplantation, which is problematic due to clinical processes, deficiency of eligible donors and rejection. In this scenario, BGs could be employed but much research is still needed. In this field, the first *in vitro* study testing BGs was conducted in 2003 in order to verify the cyto-compatibility of sol-gel 58S glass scaffolds linked with laminin or amine using MLE-12 lung cells. Results showed that 58S BG-based scaffolds presents cyto-compatibility, revealing positive effects in cell adhesion and growth (Kargozar et al., 2019). Moreover, BGs have been added to polymeric matrices in regenerative strategies relating to the respiratory epithelium; in particular, PDLLA/45S5 Bioglass® has been added to A59 lung epithelial cells *in vitro*. An increased cell adhesion, directly proportional to the bioglass content, was observed (5 wt%) (Kargozar et al., 2019). The gastrointestinal epithelium can be compromised by ulcers; treatment consists in the assumption of oral drugs to eradicate *Helicobacter pylori*, inhibit gastric acid secretion and neutralize gastric acid. Previous data suggested that 45S5 Bioglass® promotes epithelial regeneration and cell migration in the wound site, allowing the gastrointestinal epithelium injury to heal. As a result of the above mentioned *in vitro* study, wound healing was not achieved as a result of cell proliferation but by epithelial reconstitution (Moosvi and Day, 2009). Given the promising results, this study was extended *in vivo* by another research group through inducing ulcers in rats and mice and administrating 45S5 Bioglass® powder. Results suggest that glass powder had a similar effect to omeprazole and hydrotalcite, two commonly used drugs. Indeed, constant oral administration of glass powder shows a defensive role on gastric ulcers thanks to the ability of the glass to neutralize gastric acid through alkaline ion release. These data make BGs the perfect candidates for gastrointestinal tissue regeneration (Kargozar et al., 2019). Barium-containing BGs (BaBGs) were also studied recently for use in the treatment of gastro-duodenal ulcers; it seems that these BGs form a protective barrier of the gastro-duodenal epithelium and increase gastric pH, counteracting gastric acidity (Kargozar et al., 2019). Although these studies show promising results, additional investigations are need to implement BGs knowledge in the repair of gastrointestinal and respiratory epithelia. Taken together, the abovementioned studies indicate that BGs have reached a respectable level of interest as biomedical tools with a broad range of applications in soft tissue engineering. In particular, BGs have shown biocompatibility, capacity of bonding to living tissues, antibacterial and anti-inflammatory proprieties, and the ability to stimulate angiogenesis, making them perfect substitutes for conventional treatment of soft tissue injuries.

## Polymers and Composite Scaffolds for Soft Tissue Repair

Polymers are one of the most commonly used scaffolding biomaterials, possessing great manufacturing stability, biocompatibility and biodegradability. Natural polymers including chitosan, gelatin, collage, alginate, and Polyester-based polymers such as polylactide (PLA), poly (lactic-co-glycolic acid) (PLGA), polycaprolactone (PCL), poly-glycerol-sebacate (PGS), and polyurethane (PU) are used as materials for both soft (Siddiqui et al., 2018) and hard tissue repair (Iaquinta et al., 2019a). For example, PCL, a linear synthetic biodegradable aliphatic polyester, a low cost material, because of its flexibility can be employed for the realization of different scaffolds (Oh et al., 2007).

Conducting polymers (CPs) as an innovative generation of organic materials are characterized by intrinsic electrical conductivity, with good mechanical and electrical features, stability, and biocompatibility (Yuk et al., 2020). Among CPs, polypyrrole (PPY), polythiophene (PTh), and related compounds are interesting materials for biomedical applications (e.g., drug-delivery systems, artificial muscles, bioactuators, biosensors) due to their biocompatibility (Yang et al., 2016), easy synthesis and simple modification (Ravichandran et al., 2010). On the other hand, the biomedical employments of polyaniline (PAni) are questioned because of its putative cytotoxicity (Li et al., 2014; Bober et al., 2015). However, PAni has some interesting characteristics, i.e., (i) it is easily produced from common chemicals, (ii) it has high electrical conductivity, optimal electronic and optical properties, good redox and ion-exchange activity, (iii) it has environmental stability, and (iv) low cost (Visakh, 2018). CPs can be employed, with or without electrical stimulation, to improve several cellular activities including cell attachment, proliferation, migration and differentiation (Kaur et al., 2015). CPs-based biomaterials have received growing interest for engineering electrical sensitive tissue, above all cardiac muscles, nerves, skins, and bones (Guo and Ma, 2018), whereas in combination with biodegradable polymers it can be employed to produce scaffolds owing electroactive and biodegradable features (Bertuoli et al., 2019).

## Composite Biomaterials Tissue Rigeneration

### Composite Biomaterials for Epithelial Regeneration

It is known that skin is able to protect the human body from damage and against microbial invasion. Worldwide, burns represent a serious concern for the health. Approximately 260,000 patients die each year because of thermal burn wounds (Forbinake et al., 2020). Composite biomaterials have been widely employed to repair damaged skin tissue (Chaudhari et al., 2016), as well as conductive materials have also been proven to improve wound healing by promoting cellular activities including fibroblasts and keratinocytes (Guo et al., 2012; Tandon et al., 2018). In 2016, Karim et al., using double full-thickness skin wound models on the dorsum of SD rats, investigated the wound healing performance of conductive nanofiber composites based on poly (aniline-co-aminobenzene sulfonic acid), poly (vinyl alcohol) and chitosan oligossacaride. The results showed that in 2 weeks the conductive allowed almost complete healing and enhanced collagen and granulation, compared to the control group. These data indicate that the conductive nanofiber composite is an interesting product to cure wounds (Karim et al., 2016).

A recent investigation reported on PCL/PU composite porous material produced by combining salt leaching and phase separation techniques (Firoozi et al., 2017). These authors demonstrated that *in vitro* seeded fibroblasts attached and multiplied on the biomaterial suggesting the potential use of scaffolds derived from engineered skin (Firoozi et al., 2017). In another study, Ghosal et al. (2017) analyzed the surface and structural compatibility of biodegradable PCL-derived scaffolds, i.g., PCL nanofibers (PCL-NF), collagen-coated PCL nanofibers (Col-c-PCL), and titanium dioxide-incorporated PCL (TiO_2_-i-PCL) nanofibers produced employing the electrospinning method. Their results demonstrated that the addition of the TiO_2_ nanoparticles allowed to improve the mechanical strength with a decreased of cell viability. The MTT and the cell adhesion assays showed that these nanocomposites are good biomaterials for skin tissue engineering. Further investigations should be carried out in order to optimize nanoparticle concentration and to enhance the antibacterial properties of TiO_2_ with a minimization of its toxic properties.

Recently, Sharif et al. (2018) investigated a three-dimensional (3D) nanofibrous PCL/collagen scaffold using human endometrial stem cells (hEnSCs). Compared to those on bare PCL, the attachment and multiplication of hEnSCs on the PCL/collagen biomaterial were enhanced. Therefore, hEnSCs, particularly when grown on PCL/collagen nanofiber scaffolds, are novel sources of stem cells to be employed *in vitro* for skin tissue regrowth. Therefore, as a temporary skin replacement, the use of hEnSCs for skin regrowth can be considered a new therapeutic approach.

Different hyaluronic acid-based materials have tissue healing applications. In an experimental rat model it has been shown that composite PU foams impregnated with hyaluronic acid and silver sulfadiazine can significantly reduce wound size (Cho et al., 2002). Other composite hydrogels based on hyaluronic acid can be designed for tissue repair application by either cross-linking hyaluronic acid with glyceryl methacrylate groups and DNA or functionalization of hyaluronic acid with thiol cross-link sites to form a stable hydrogel scaffold (Eng et al., 2010; Chen, 2012).

### Composite Biomaterials for Nervous Tissue Regeneration

In recent years, the quality of life has been notably reduced in patients affected by nerve injury due to trauma and neurodegenerative diseases. Indeed, several nerve injuries occur daily, whereas total numbers are increasing every year. During nerve regeneration, the biomaterial employed for nerve tissue repair should be tubular, flexible and mechanically stable (Siddiqui et al., 2018). In order to reconstruct damaged nerve tissues, a comparative investigation on biomaterials produced with PCL, PCL/collagen and nanocomposites of polymer blend of PCL/Collagen/NBG by electrospinning technique was carried (Mohammadi et al., 2017). The MTT assay assessed the biocompatibility, cell viability and multiplication in 3D culture of PCL/collagen/NAB material. This result suggests that these type of scaffolds are suitable for peripheral nerve tissue repair (Mohammadi et al., 2017). Shafei et al. (2017) reported a new processing approach to realize electroactive nanofibers. By electrospinning and vapor phase polymerization, electrically conducting, robust nanofibers including both a biodegradable component using PCL and a conducting component, PPy, have been developed. In particular, the morphology, electrical conductivity, and dimensional stability of the PCL/PPy nanofibers were studied. Results suggested high potential levels in the conducting materials for neural tissue repair. It has been demonstrated that electrical stimulation can be an effective approach for neuronal function and nerve regeneration (Zhang et al., 2007). PC12 cells have been reported to display an increased percentage of neurite-bearing cells when seeded on conductive PPY/PDLLA conduits and when 100 mV electrical stimulation is applied for 2 h. Moreover, there was an increase of median neurite length with the increase of PPY content. The PPY/PDLLA nerve conduit with 5% PPY was employed *in vivo* to fill a 10 mm defect in the sciatic nerve of a rat. The results showed that it showed functional recovery like to the autologous nerve graft and significant improvement respect to the PDLLA conduits, demonstrating its great potential for regeneration of nerve tissue (Xu et al., 2014). In order to develop a suitable scaffold for Schwann cell nerve tissue engineering Song et al. (2018) analyzed three kinds of scaffolds, including pristine collagen, pure oxidized regenerated cellulose-Ca (ORC-Ca), and collagen/ORC-Ca composite scaffolds. Experimental data showed that collagen/ORC-Ca composite is a promising scaffold, which enable peripheral nervous repair and regeneration.

### Composite Biomaterials for Cardiac Tissue Regeneration

Cardiovascular dysfunctions are associated with the highest mortality in the world. Commercial vascular grafts are often made of synthetic polyester material, designed to repair damaged blood vessels. In recent times, various devices made of PCL such as vascular grafts, stent materials, and valves have gained much attention because of the possibility of molding it into knitted and woven forms in cardiac surgery (Ahmed, 2013). In this contest, PCL has been blended with a novel biodegradable electroactive PU containing aniline pentamer (AP). A solution of poly (ethylene glycol) and salt particles, in a double porogen particulate leaching and compression molding technology was developed, then the prepared blend (PB) and PCL were further fabricated into scaffolds. In this instance, scaffolds were able to favor adhesion and development of neonatal cardiomyocytes, with PB having an enhanced role on heart genes expression related to the (i) muscle contraction and relaxation, (ii) alignment of cytoskeleton proteins, troponin-T and actinin-4.

Baheiraei et al. suggested the possibility of using AP as an electroactive moiety to induce cardiomyocyte multiplication with the aim to heal/replace cardiac tissue/cells (Baheiraei et al., 2015). Liu et al. (2018a) investigated the properties of engineered spherical TiO_2_ in poly-ethylene glycol (PEG) and chitosan hydrogel matrixes. In this investigation, addressed to heart cell regrowth, patches with high bioactive and mechanical characteristics were employed.

In order to stimulate cardiomyocyte activity and myocardial performance after infarction, Wang et al., incorporated calcium silicate into the controllable aligned chitosan electrospun nanofibers (Wang et al., 2019). Their results showed that Si ions favored the heart-specific genes expression and neonatal rat cardiomyocytes multiplication, suggesting a potential application for bioactive ions and nanostructured biomaterials in cardiac tissue engineering.

In post myocardial infarction, the employment of MSCs transplanted in heart seems to a potential approach to repair cardiac tissue/cells. Specifically, it was highlighted that the paracrine effects of transplanted MSCs play a pivotal role in cardiac regeneration through the secretion of many growth factors and immune-related cytokines. At present, MSCs employed for heart repair did not work at high efficiency as expected. This approach is limited by the poor viability and activity of transplanted MSCs (Iaquinta et al., 2019b). In addition, negative parameters for transplanted stem cells are represented by different stresses, such as high shear, oxidative environment, the immune reaction of the host. To circumvent these negative reactivities, it has been suggested that stem cells could be encapsulated by electro-spraying in microgels composed by graphene oxide (GO)/alginate (Choe et al., 2019). In treating different injuries and diseases, like MI, the authors proposed this delivery system for stem cells or other therapeutic cell types.

## Hydrogels in Soft Tissue Engineering

**Figure 3:**
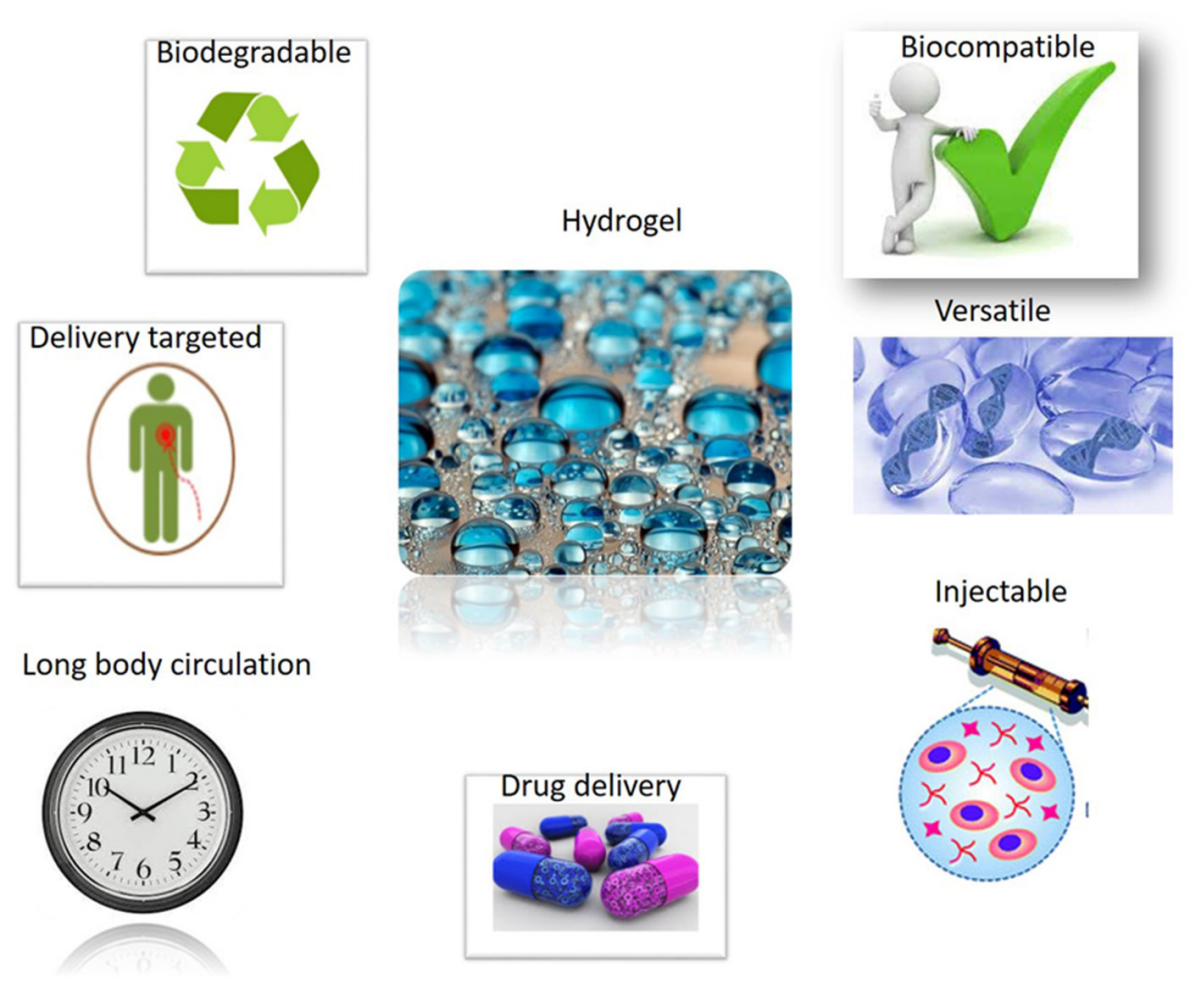
Successful applications of Hydrogel materials will benefit from their attractive properties. Hydrogel materials own good biocompatible and delivery proprieties. Hydrogels show a long body circulation, with a versatile use including excellent injection capabilities. Hydrogels are biodegradable materials, too.

Most tissue engineering approaches employ stem cell encapsulation within highly porous, natural-like biomaterials/scaffolds for tissue regeneration and repair (Iaquinta et al., 2019b). Among different biomaterials currently used in tissue engineering, hydrogels are considered very promising (Talebian et al., 2019) (Figure 3).

The term hydrogel and the underlying concept were first used in 1894, when Bemmelen, described the effect of water absorption on copper particles (Bemmelen, 1906).

Hydrogels are defined as a three-dimensional network capable of retaining many molecules of water avoiding to become solvated (Vashist et al., 2014). They are classified as soft materials and their origin can be natural and/or synthetic. Those of a synthetic nature include several hydrogels e.g., polyacrylamide pNIPAAM (poly-N-isopropylacrylamide), PVA (polyvinyl alcohol), or PVP (polyvinylpyrrolidone). Hydrogels of natural origin are: chitosan, collagen, gelatin, dextran, cellulose, and hyaluronic acid (Gaharwar et al., 2014; Biondi et al., 2015; Caló and Khutoryanskiy, 2015; Ullah et al., 2015).

Due to their specific characteristics, hydrogels are of particular interest for biomedical applications (Biondi et al., 2015; Mondal et al., 2020). Specifically, the use of hydrogels have been suggested in regenerative medicine, tissue engineering, and controlled drug delivery, thanks to their biological, chemical and physical proprieties, which are compatible with those of biological tissues (Talebian et al., 2019; Mondal et al., 2020). There are different classifications of hydrogels in literature. Hydrogels (HGs) are classified on the basis of their physical features, the swelling property, production process, provenience, ions, derivative, bio-recycling, and cross-linking activities (Qiu and Park, 2012). Depending on the cross-linking agent, there are physical or chemical hydrogels. In physical gels, the crosslinking process has a physical nature. Physical processes, such as hydrophobic association, crystallization, polymer chain complexity and hydrogen bonding allow this kind of cross-linking to be obtained. Nonetheless, a chemical process, i.e., chemical covalent crosslinking, prepares chemical hydrogels. Chemical hydrogel, differently from physical hydrogels, are permanent and irreversible due to their configurationally changes. Based on bound group charges, HGs can be classified as neutral, cationic or anionic. Hydrogels can also be divided based on their structure, specifically they can be amorphous, semi-crystalline, crystalline or hydro colloid aggregates (Ullah et al., 2015). It is possible to obtain another category of hydrogels, called double-network hydrogels (Nonoyama and Gong, 2015). Specifically, these HGs are composed, by combining their physical and chemical characteristics, of cross-linked HDs by electrostatic interaction.

Another important class of hydrogels is constituted by stimuli-responsive hydrogels, which are able to transform stresses, such as magnetic, optical, chemical, electrical, mechanical stimuli, into a different kind of message/signal. They can respond to environmental stimuli by variations in their growth actions, network organization, permeability and mechanical strength. For this reason, they are called environmentally sensitive, smart hydrogels (Gil and Hudson, 2004; Kang et al., 2013; Ullah et al., 2015). Physical stresses can be represented by light, pressure, temperature, electric and magnetic, mechanical stimuli and the intensity of several energy sources. Ionic factors, pH and chemical agents are included in chemical stimuli (Kang et al., 2013). Instances of stimuli responsive gels, which respond to temperature, are chitosan-based hydrogels, i.e., methylcellulose, hydroxyl-propyl-methyl-cellulose and Nisopropylacrylamide (NIPAAm). The most highly-studied is poly (N-isopropylacrylamide) (PNIPAAM), which is capable of generating chemical or mechanical signals at different temperatures. Indeed, below a critical temperature, called the lower critical temperature of the solution (LCST), this hydrogel shows hydrophilic characteristics because of the hydrogen bonds which link pendant groups and H_2_O molecules. A temperature higher than the LCST causes the bond to break and makes the polymer hydrophobic (Ekerdt et al., 2018; Han et al., 2018). Bossard et al. reported that polydiethylaminoethyl methacrylate (PDEAEMA) and its copolymers have the ability to ionize in response to pH (Bossard et al., 2006). Diversely, in a work conducted by Dolatabadi et al., they reported that their hydrogel composed of alginate-N, O-carboxymethyl chitosan (NOCC) gel beads coated with chitosan is pH sensitive. Indeed, their investigations indicated that the swelling property at pH 7.4 is increased compared to pH 1.2 (Dolatabadi Farahani et al., 2006). Hydrogels employed as scaffolds in regenerative medicine are produced with pore size in which cells can be seed and grow. In addition, HGs are designed to dissolve/degrade in a way (i) to release growth factors and (ii) to generate pores hosting proliferating cells (Hoffman, 2012). In comparison to other biomaterials derived from metallic/ceramic scaffolds, HGs due to their characteristics can be designed with similarities to soft-tissues (Wang et al., 2018).

Advances in hydrogel technology have allowed a new category of hydrogels to be developed called nanocomposite hydrogels (NCHs). NCHs are highly hydrated polymers which form networks able to interact with a 3D structure. This structure retains water if containing nanoparticles/nanostructures covalently/non-covalently bond to the matrix. Several polymers, both synthetic and naturally derived, can be used to make the hydrogel, which can be used to incorporate many different nanomaterials. These NCHs that are produced can be used for various biomedical applications. Many different nano-particulate systems can be included in the hydrogel network to obtain NCH. Specifically, carbon-based nanomaterials, and polymer/ceramic/metal/metal oxide nanoparticles can be used. The addition of specific nanoparticles can strengthen the initial HGs and provide NCHs with reactivity to external stimuli. In turn, NCH react to stimuli determined by the nature of the nano-systems incorporated in the hydrogel (Annabi et al., 2014; Biondi et al., 2015).

Carbon nanotubes (CNTs) and graphene, as well as other carbon-materials, are employed to improve the mechanical and electrical properties of conventional hydrogels thanks to their high electrical conductivity (Biondi et al., 2015). To date, both CNTs- or graphene-based NCHs have been investigated for their potential use in different biomedical applications, i.e., biosensors, scaffolds, drug vectors, and biomedical devices (Cha et al., 2013; Goenka et al., 2014; Qu et al., 2018; Saleem et al., 2018; Maiti et al., 2019). There are distinct atomic set up of CNTs, such as armchair and zig-zag, and designs, as single- and multi-walled. In addition these CNTs can be chanced in their chemistry to enhance hydrophilicity/interaction with HGs (Cha et al., 2013).

NCHs incorporating carbon-based nanomaterials can potentially be used, due to their high electrical conductivity, in several tissues, including nerves, muscles and heart, and for electrically stimulated drug delivery (Mottaghitalab et al., 2013; Qu et al., 2018; Saleem et al., 2018; Mondal et al., 2020).

Nanocomposite hydrogels include NCHs based on polymeric nanoparticles. The main objective for including particles in the hydrogels is (i) to increase the mechanical properties of hydrogels, (ii) to ensure drug release in a controlled manner, and (iii) to provide mechanical reinforcement. Furthermore, to facilitate the solubility of poorly soluble drugs, polymeric nanoparticles are designed with amphiphilic macro-molecules, such as micelles, dendrimers and hyper-branched polymers (Biondi et al., 2015). NCHs incorporating polymeric nanoparticles can help to load hydrophobic drugs in hydrogels. Different chemotherapeutic drugs have low solubility in water, potentially causing unwanted noxious side effects. In their study, Ju et al. loaded paclitaxel within a thermos-sensitive micelle/HG hybrid system derived from Pluronic F-127 and carboxymethyl chitosan. The aim of this study was to avoid the drug danger and to enhance its water solubility. This model was cross-linked with glutaraldehyde and developed for local chemotherapy. The *in vivo* studies reported revealed that tumor progression rate was decreased and side effects reduced compared to free paclitaxel (Ju et al., 2013).

A HG material produced from a tri-block copolymer compound with a PEG core and methacrylated poly (glycerol succinic acid) dendrimer terminal block has been synthesized for soft tissue applications. *In vitro* studies have reported that the NCH induced significant synthesis of neo-cartilaginous material. Indeed, encapsulated chondrocytes synthesized new cartilage-like structure with proteoglycans and type II collagen protein (Söntjens et al., 2006).

Another feature of these NCHs is that they are optically transparent in both wet and dry states. This also makes them promising for applications such as optical devices (Ghobril et al., 2016).

A new class of biomaterials is composed of based on metal and metal oxide nanoparticles. These NCHs have different characteristics, which own antimicrobial activity, together with reactivity to electrical/magnetic/light stimuli. These nanoparticles include metals, such as Au, Pt, Ag, Co, and Ni. Other metal nanoparticles are designed with iron oxide (Fe_3_O_4_, Fe_2_O_3_), titania (TiO_2_), alumina (Al_2_O_3_), and zirconia (ZrO_2_) (Ghobril et al., 2016; Wahid et al., 2017). Metal and metal oxide nanoparticles provide antimicrobial activity for NCHs, as they bind non-specifically to bacterial membranes, thereby inducing structural alterations in bacteria, by enhancing the membrane permeability. Furthermore, metal nanoparticles because of their ferromagnetic and conductive/semiconductive characteristics provide NCHs with electrical and magnetic capabilities suitable for applications in biomedicine field, such as tissue regeneration (Wahid et al., 2017; Zhang et al., 2019). NCHs with silver nanoparticles are among the most promising of those with an antimicrobial effect. The functional coating property of Ag-based NCHs have been employed in odontoiatric applications (dental filling) and to prevent skin infections as a dressing in wound and burn. NCHs have demonstrated the capacity to be designed as heart patches for the treatment of damaged heart tissue after a heart attack (Wang et al., 2015, 2017; Shao et al., 2017; Wahid et al., 2017; Liu et al., 2018b; Solazzo et al., 2019). Additionally, metallic or metal-oxide based nanocomposite hydrogels could also be used for different purposes, such as bioactivation, biosensing, diagnostic activities, delivery and release of distinct therapeutic compounds (Biondi et al., 2015).

Nanocomposite hydrogels, together with other methods, can be produced combining inorganic ceramic nanoparticles with hydrogels, which are composed of different polymers, either synthetic or natural. To this purpose, several bioactive nanoparticles, including HA, synthetic silicate nanoparticles, bioactive glasses, silica, calcium phosphate, glass ceramic, and b-wollastonite, can be employed. Thanks to their great mechanical resistance, ceramic nanoparticles can strengthen hydrogels. In addition, these nanoparticles are useful because the final NCH product acquires biological characteristics. Indeed, minerals in the hydrogel give significant characteristics, improving in human tissues their homeostasis and regeneration (North, 2002; Gaharwar et al., 2014). These properties make such nanoparticles excellent candidates for their employment in regenerative medicine and tissue engineering. Among different scaffolds available, HA-based hydrogels are the most widely used. HA is a naturally occurring linear polysaccharide that makes up the ECM in human connective tissues. In order to favor the physical property of HA scaffolds, HA-based hydrogels have been strengthen with different types of nanoparticles, such as calcium and silica (Burdick and Prestwich, 2011). Another widely used natural polymer is chitosan. Indeed, chitosan has biocompatibility, biodegradability, non-toxicity, and cicatrisation properties (Pellá et al., 2018; Xu et al., 2018; Khan et al., 2020). Conversely, the most widely-used synthetic material in TE is PEG thanks to its recognized biocompatibility. All these polymers can be reinforced with ceramic materials to improve their performance. Chen et al., in their study developed a new composite nano-fibrose-gelatin/bioactive glass hydrogel (NF-GEL/BG) using the phase separation method, followed by reinforcing nanofibers with hyaluronic-chitosan pairs. This compound improved its microstructural and thermal integrity. Therefore, this bioactive hydrogel may offer close biomimicry to the fibrous nanostructure, facilitating in natural soft tissue components the treatment of non-healing wounds (Chen et al., 2013).

## Scaffold to Treat the Hard-Tissue Interface in Ligament/Tendon- to-Bone Junction

The heal process of ligament/tendon-to-bone-junction is still a difficult challenge in the orthopedic filed medicine, being the main problems to be solve the poor vascularity and multi-tissue transitional structure of the junction. The tendon/ligament-to-bone interface is difficult to repair, whereas the functional graft integration after injury not always occurs. Many different approaches have been attempted to favor the healing process of the ligament-bone junction. *In vivo* experiments/clinical trials employing stem cells, bioactive factors, and synthetic materials have been carried out. However, these approaches did not repair the complex structure-function interactions among different tissues. *In vivo* experiments carried out in rabbit, in its anterior crucial ligament reconstruction showed that the modified Random-Aligned-Random material increased bone and fibrocartilage formation in the interface. This result seems to be better than the unmodified tendon ECM (Liu et al., 2017).

The heal process of injured tendon is a difficult task. Indeed, high rates of relapse due to the stress induced by scar formation and sutures employed during surgery has been recorded. A recent investigation reported the tendon-to-bone interface regeneration employing a gradient material produced by a one-station electrospinning process, with osteosarcoma-like cells and fibroblasts grown on a PLC nanofiber scaffold (Nowlin et al., 2018). The microstructure of the biomaterial seems to be a possible clue for mimicking transitional interface between different tissues. This model, representing a heterogeneous cellular composition platform which may favor the regrowth of multi-tissue complex systems, deserves additional studies (Nowlin et al., 2018).

## Biomaterials as Drugsdelivery System

Biomaterials seem to be a good model of study for *in situ* drug delivery. Their use may avoid at the same time peri-implant traumas, while favoring tissue regrowth.

HA formation on produced pollen-templated bioactive glass particles (PBGPs) with tetracycline hydrochloride showed dual macro-nano porous construction. *In vitro* experiments with macro-nano porous PBGPs showed non-cytotoxic effects in Hep G2 cells, a human hepatocellular carcinoma derived cell line, after co-culture for up to 72 h. These data indicated that the biocompatible hierarchically macro–nano porous PBGPs could be an interesting strategy to be employed for local drug delivery applications (Zheng et al., 2015). Such materials can also works as carriers for drugs delivery to care musculoskeletal infections and to avoid any systemic toxicity (Köse et al., 2018).

Since about 70 years, bioceramics have been employed for tissue renewal. Several synthetic bioceramics were developed for biomedical applications. Recent advances in nano-science and -technology, indicate that bioceramics can be employed to develop innovative *in situ* drug delivery models (Vallet-Regí, 2019). In fact, bioceramics have been already investigated for their carrier suitability in drug delivery for antibiotics, anti-osteoporotic and anticancer compounds.

It is advisable, in medicine, to administer therapeutic agents that do not alter the physiological system.

However, in many cases, the dosages of prescribed drugs are higher than necessary, but they are needed to ensure the efficacy of the drugs in affected area. In this context, it should be recalled that drug doses administered to the patient, because act systemically, affect not interested regions.

The use of bioceramics for local drug delivery is indicated because they allow a specific *in situ* drug delivery without side effects. Direct drug administrations to the site overcome the potential systemic toxicity. Bioceramic nanoparticles represent favorable carriers, which are alternatives to polymers, because of their characteristics. Indeed, they are bioactive, with pH and temperature stability, multi-functionality, biocompatibility, and tunable biodegradability. The nanoparticles are designed for drug delivery to care serious diseases such as cancer. In this kind of treatment, the nanoparticles linked with therapeutic drugs are directed to the affected area where the agents are to be released.

However, in this case, a highly cytotoxic drug is transported, so premature release into tissues other than the tumor must be avoided. For this reason, stimuli-responsive systems have been developed. However, it is very difficult to design completely safe systems in which no release occurs before reaching the target. As an alternative strategy, nanocarriers could be loaded with cytotoxic agents in an inactive state. When the nanocarriers reach the target tissue, they are activated so that highly toxic compounds are generated only in the target tissue. In this way, nanoparticles capable of carrying a prodrug were developed which could be activated by specific enzymes overexpressed in tumor tissue (Bildstein et al., 2011). This strategy has a great limitation, as the concentration of activating enzymes in the tumor is very low or even the total absence of natural enzymes capable of activating certain pro-drugs. Bioceramics can also be applied as a vector for drug delivery in musculoskeletal infections without causing any systemic toxicity (Köse et al., 2018). An investigation reported the development of a double local drug delivery system (tetracycline and ibuprofen) composed of bioceramic calcium phosphate nanocarriers. This recent model was developed with the aim at treating the antibacterial, anti-inflammatory and bone regenerative aspects of periodontitis. *In vitro* and *in vivo* results suggest that the combined drug delivery platform can provide comprehensive management for all bone infections requiring multiple drugs (Madhumathi et al., 2018). Another system of multidrug delivery is represented by a calcium sulfate hemihydrate (cSH)/nanoHA based nano-cement (NC) biomaterial employed as a local delivery carrier for the two standards of tuberculosis care drugs, rifampicin and isoniazid. Long term multiple systemic antibiotics form the cornerstone in the treatment of bone and joint tuberculosis, often combined with local surgical eradication. These findings suggest that a biphasic ceramic based drug delivery system could be a promising alternative treatment for bone and joint tuberculosis (Qayoom et al., 2020). Indeed, bioceramics carrier systems are employed in the local delivery of growth factors, representing a promising and innovative tool in tissue engineering and dentistry.

In a study, conducted by Kovacevic and co-workers, the local delivery of TGF-b3 has been tested as an injectable calcium-phosphate (CaP) matrix to the healing tendon-bone interface after rotator cuff repair in a rat model. It was demonstrated that the treatment with TGF-b3 delivered with an injectable Ca-P matrix can strengthen the healing enthesis, promote bone regeneration, increase collagen organization, and decrease scar formation compared with repair alone in a critical damage and repair model. In addition, it has been reported that the CaP matrix by itself can promote early bone formation, increase area of fibrocartilage, and increase collagen organization at the healing enthesis (Kovacevic et al., 2011). Interestingly, the use of injectable CaP cement combined with enamel matrix derivative (EMD) was assessed in an *in vivo* study, to evaluate ligament tissues regeneration and bone formation in the periodontium. In this study, the combination of EMD and CaP cement produced the most favorable effects for periodontium formation and bone regeneration, respectively. For this reason, the use of CaP in combination with EMD could be a promising treatment for the regeneration of ligament and bone tissues in the periodontium (Oortgiesen et al., 2013). Among the different scaffolds used as drug delivery systems, CaP cements are also studied. Takayuki et al. evaluated the use of CaP cement loaded with doxorubicin as a scaffold capable of releasing the drug and filling a postoperative defect. The results of this study showed that the drug-loaded Ca-P cement suppressed the proliferation of RMT-1 E4 rat breast cancer cells. Additionally, the same material loaded with doxorubicin significantly inhibited the proliferation of sarcoma 180 cells in the mouse airbag model. These results indicate that CaP cement with doxorubicin could be applied in the local treatment of malignant soft tissue tumors (Tani et al., 2006).

The CaP cement scaffolds were also loaded with vascular endothelial growth factor (VEGF). A recent study reports the method of fabrication of a CaP cement scaffold loaded with VEGF using the 3D plotting system. In this work, the functionalized scaffold was found to be cyto-compatible and maintained the bioactivity of VEGF during the fabrication procedure (Akkineni et al., 2015).

## Conclusions

It is known that a large part of the human body is composed by soft tissues. They form, play pivotal roles in maintaining both the structure and function of different organs. At present repair and regeneration of injured soft tissues (e.g., skin, peripheral nerve, cardiac tissue, etc.) remains a great challenge in regenerative medicine. Different types of bioceramics, including bioactive glasses, carbon nanostructures, and HA nanoparticles are proposed as innovative biomaterials for healing/regrowth of many different soft tissues. This review has been written with the aim to draw the attention of the readers interested to know the state of the art in soft tissue repair and potential employments of bioceramics in this regenerative medicine field.

## Author Contributions

EM, MI, CL, CM, and MMa collected the literature, designed the figures, and wrote the draft of the manuscript. SS, MMo, and AT edited the manuscript format and corrected the draft. FM and MT revised the manuscript and edited the final version. CL is a fellow recipient of the European Project High skills for research and technological transfer, from the Regione Emilia Romagna, POR FSE 2014/2020. All the authors contributed to the article and approved the submitted version.

## Conflict of Interest

The authors declare that the research was conducted in the absence of any commercial or financial relationships that could be construed as a potential conflict of interest.
